# Utilization of a cardiometabolic health nurse – a novel strategy to manage comorbid physical and mental illness

**DOI:** 10.15256/joc.2014.4.36

**Published:** 2014-07-24

**Authors:** Brenda Happell, Robert Stanton, David Scott

**Affiliations:** ^1^School of Nursing and Midwifery, Centre for Mental Health Nursing Innovation, Institute for Health and Social Science Research, Central Queensland University, Rockhampton, Queensland, Australia; ^2^NorthWest Academic Centre, University of Melbourne, Melbourne, Victoria, Australia

**Keywords:** cardiometabolic health nurse, comorbid conditions, mental health, patient referral, physical health

## Abstract

**Background:**

Comorbid chronic illnesses, such as cardiovascular disease, respiratory conditions, and type 2 diabetes are common among people with serious mental illness. Management of comorbid illness in the mental health setting is sometimes ad hoc and poorly delivered. Use of a cardiometabolic health nurse (CHN) is proposed as one strategy to improve the delivery of physical health care to this vulnerable population.

**Objective:**

To report the CHN’s utilization of primary care and allied health referrals from a trial carried out in a regional community mental health service.

**Design:**

Feasibility study. Mental health consumers were referred by their case manager or mental health nurse to the CHN. The CHN coordinated the physical health care of community-based mental health consumers by identifying the need for, and providing referrals to, additional services, including primary care, allied health, and community-based services.

**Results:**

Sixty-two percent of participants referred to the CHN received referrals for primary care, allied health, and community-based services. Almost all referrals received follow-up by the CHN. Referrals were most commonly directed to a general practitioner and for nurse-delivered services.

**Conclusion:**

The CHN role shows promise in coordinating the physical health of community-based mental health consumers. More studies on role integration and development of specific outcome measurement tools are needed.

Journal of Comorbidity 2014;4:22–28

## Introduction

People with serious mental illness (SMI), particularly psychotic disorders, are known to exhibit poor health behaviors [1] and experience significant healthcare disparities [2, 3]. Taken together, this contributes to a significantly greater risk of chronic comorbid physical health conditions, compared with the general population [4, 5]. As a result, people with SMI experience premature death up to 25 years earlier, compared with the general population [6, 7].

Nurses who work in mental health are acutely aware of the poor physical health of mental health consumers, and the need for routine screening and monitoring [8]. Mental health consumers are also aware of the complexities associated with the management of their physical health, and recognize the need for flexible solutions to address the gaps in primary and secondary healthcare services [9].

Unfortunately, there are significant biopsychosocial barriers to improving the physical health of mental health consumers, including challenges with consumer engagement, medication side effects, and low motivation [10]. These barriers are compounded by organizational policies and practices, such as role descriptions and lack of information sharing, which fail to support the physical health needs of consumers [11, 12]. Nurses also report being limited in their capacity to provide physical health care due to time and resource limitations [13, 14].

Despite these barriers, there are nurse-led interventions, which have shown promise in improving the physical health and reducing cardiometabolic risk in mental health consumers [15–17]. A common theme of these interventions has been the integration of primary care and mental health services, coupled with the use of comprehensive screening procedures for the detection of cardiometabolic risk factors. This is consistent with the recommendations of De Hert and colleagues [18], and recent evidence suggests these and other interventions are well supported by nurses [19].

Of particular importance in the success of interventions targeting improvements in the physical health of people with SMI is the referral to, and follow-up from, primary care service providers. Failure to address this critical step is unlikely to result in significant advancement of the physical health of people with SMI, and may increase the already widening service gap. To address the systemic barrier of role designation, a specialist nursing position, namely the Cardiometabolic Health Nurse (CHN) has been proposed [20]. Given the prevalence of comorbid illness and the need to address the gaps in screening for cardiometabolic health risk factors, we recently conducted a 26-week feasibility trial of a specialist CHN in a regional mental health service in Queensland, Australia. The purpose of this paper is two-fold. Firstly, to identify self-reported chronic illness and risk factors for chronic illness in consumers attending a CHN clinic. Secondly, to assess the utilization of referrals to primary care, allied health, and community-based healthcare services to address the comorbid physical health of community-based mental health consumers attending the CHN clinic.

## Methods

The protocol for the CHN trial has been previously published [21]. Briefly, a registered nurse with more than 10 years of experience in community and inpatient mental health settings was employed as a CHN in the mental health service for 26 weeks. The goal of the CHN was to coordinate the physical health care of community-based mental health consumers by identifying the need for, and providing referrals to, additional services, including primary care, allied health, and community-based services.

### Setting

The trial was conducted in a regional community mental health service in Queensland, Australia. The service has more than 400 people registered as users and receives around 85 referrals each month.

### Participants and recruitment

All English-speaking, community-based adult mental health consumers attending the mental health service during the recruitment period were invited to participate in the study by their consultant, case manager, or nurse. Potential participants were asked during the course of a usual consultation if they would be interested in participating in the study. Those who expressed an interest were offered an information sheet describing, in plain language, the aims of the study, and what participation would involve. Participants who agreed to participate were then provided with an informed consent form and contact with the CHN was arranged. On receipt of the signed informed consent form, the CHN then contacted the participant to arrange an initial appointment. Organizational support for the recruitment of consumers to the study was provided by the Director of Nursing.

### Data collection

Baseline data collection comprised identification of comorbid cardiometabolic risk factors, such as hypertension and obesity, and at-risk behaviors, including assessment of physical activity (Active Australia Survey [22]) self-reported intake of fruit and vegetables [23], nicotine dependence (Revised Fagestrom Tolerance Questionnaire [24]) and alcohol misuse (Alcohol Use Disorders Identification Test [25]). All data pertaining to the measures used in the study protocol were collected by the CHN during the course of the consultations. Training on the use of all questionnaires was provided by the research team, comprising a Professor of Mental Health Nursing (B.H.) and Accredited Exercise Physiologist (R.S.), who were both familiar with the use of the relevant instruments.

The CHN conducted consultations using a framework based on the Integrated Theory of Health Behavior Change [26], which posits that while increased knowledge alone is insufficient to effect behavior change, increased knowledge and beliefs may positively impact on self-efficacy and self-regulatory skills, leading to positive behavior change. During the consultation, the CHN examined the health concerns and priorities of the consumer, then provided education, goal-setting, and self-management skills to meet the needs of the consumer. Taking into consideration the identified cardiometabolic risk factors or modifiable behaviors, the CHN negotiated an action plan, including referral to primary care, allied health, or community-based healthcare providers, as necessary. Guidance in the delivery of health promotion and instruction on the use of outcome questionnaires was provided by the research team. For each consumer who attended a baseline assessment, the number of referrals, the primary care, allied health, or community-based healthcare provider to whom the referral was made, and the purpose and outcome of the referral was recorded in the consumer’s file. The CHN was also required to record incentives for and barriers to positive lifestyle change noted by the consumer. To determine the use of primary care referral pathways, a file audit of participant records maintained by the CHN was carried out by a trained, experienced research assistant.

### Data analysis

Descriptive statistics were used to present the demographic characteristics of the participants. Outcomes for primary care, allied health, and community-based healthcare provider referrals are presented as frequencies. All analyses were performed using Statistical Package for the Social Sciences Version 20 (IBM Corp, NY). To avoid replication of already published data, the results of physical health behaviors are not reported in this paper as they are reported elsewhere [21].

### Ethics approval

This study was approved by the health service and University Human Research Ethics Committees. All participants provided written informed consent prior to participation and were informed that participation was voluntary and that non-participation would not impact upon treatment from the health service.

## Results

### Participants

Twenty-one community-based mental health consumers attended initial appointments for the collection of baseline data. The majority were male (61.9%), single (66.7%), and receiving a pension (80%). Educational attainment was mainly confined to secondary school level (year 12) (85.7%) with one having a lower level of education and two having technical school or university level of education.

### Self-reported chronic illness and presence of risk factors for chronic illness

Chronic comorbid physical health conditions were self-reported by 85.7% (*n*=18) of participants. Of these, 72.2% (*n*=13) reported multiple conditions (range 2–6 conditions). A substantial proportion (85.7%) of the participants were overweight or obese (body mass index ≥25.0 kg/m^2^). Waist circumference data were not available for all participants; however, for participants who consented to waist circumference measurement, 72.7% (*n*=8) recorded a waist circumference in excess of gender-specific cut-off points for increased cardiometabolic risk (females, 88 cm; males, 100 cm). At baseline screening, 42.9% (*n*=9) of participants were identified as hypertensive (systolic blood pressure >135 mmHg or diastolic blood pressure >85 mmHg).

### Referrals for primary care services

In total, 13 participants received 22 referrals to five different healthcare providers. Six participants received referrals to only one healthcare provider, five participants received referrals to two healthcare providers, while the remaining two participants received referrals to three health professionals. The majority of these were made to a general practitioner (GP; *n*=8) and for nurse-led services (*n*=9). A summary of these referrals is shown in Table 1.

**Figure 1:**
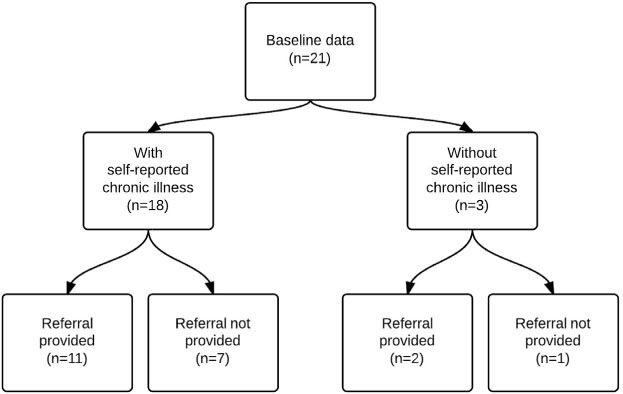
Distribution of referrals for those with and without self-reported chronic comorbid conditions.

Eleven consumers with self-reported chronic illness (61.1%) received referrals for primary care, allied health, or community-based healthcare services. Overall, eight participants, of whom seven self-reported at least one pre-existing chronic comorbid health condition, were not referred to additional health services. Figure 1 shows the distribution of referrals for those with and without self-reported chronic comorbid conditions.

From the 13 consumers offered referrals, file review identified follow-up for 12 referrals. From most files, it was not clear if the consumer made the appointment for the referral, attended the appointment, or had any specific outcomes. From the 21 participants who attended baseline assessments, 11 returned for follow-up assessment at 26 weeks. Review of consumers’ files indicates that additional referrals were made for two of these consumers. In both cases, referrals were made to the same healthcare providers recorded at the initial consultation.

The outcomes from all referrals could not be determined from the available data. However, from referrals to GPs, improved medication management was evident in two cases. From referrals to nurse-led services, advice to increase physical activity and increase daily consumption of fruits and vegetables was commonly described. Although we tried to collect data on incentives and barriers, very little information about what helped or hindered consumers in making healthy lifestyle changes was recorded in the medical records.

## Discussion

The purpose of this paper was to examine the health problems experienced by a small group of community-based mental health consumers, and to investigate the feasibility of utilizing a CHN, embedded within the mental health service setting, to coordinate their physical health care. Specifically, we report the utilization of referrals to primary care, allied health, or community-based healthcare services, to address the comorbid physical health of community-based mental health consumers attending the CHN clinic. Consistent with previous studies, a significant proportion of participants in the present study were overweight or obese [27, 28], and had a high prevalence of chronic comorbid illness [4, 29]. Almost all participants in the present study self-reported having at least one chronic illness and one participant reported six separate chronic comorbid conditions. This is consistent with the plethora of cross-sectional studies reporting the high prevalence of chronic comorbid physical health conditions among mental health consumers [4, 30, 31]. This strongly suggests that, despite the effectiveness of short-term interventions [32] to improve the physical health of mental health consumers, and integration of routine metabolic monitoring programmes into some mental healthcare programmes [33], the physical health of mental health consumers remains well below the standard of the general population.

Our data illustrate the ways that CHNs who are part of mental health programmes can be helpful in referring consumers with SMI to primary care, allied health, and community-based services. Almost two-thirds of consumers who attended CHN appointments received referrals for physical health services, and almost half of these were for multiple services. This suggests that utilization of a CHN may be a potentially useful strategy in coordinating the physical health care of mental health consumers. It is disappointing, however, that data pertaining to follow-up were only available from a small number of consumer files. This highlights the complex care needs of mental health consumers and underpins the need for coordinated, multidisciplinary healthcare teams [34, 35]. This is particularly important for the 85% of our cohort with risk factors for, or diagnosis of, comorbid chronic health conditions.

Although previous studies show improved primary healthcare linkages as a result of nurse-led interventions [15, 16], almost half the mental health consumers in the present study did not receive referrals to primary care services. As our CHN did not record the reasons for not referring consumers, we are only able to speculate why some participants in the present study were not referred for additional primary care services, despite the presence of at least one chronic comorbid health condition. It is possible that the consumer was offered, yet refused, the referral for additional healthcare services. It is also possible that the consumer was already receiving adequate treatment for the condition, and did not believe additional healthcare services were necessary. Alternatively, the consumer may not have given the condition any priority in the context of their overall health. This latter notion is supported by Buhagiar and colleagues [36] who reported that, compared with people without mental illness, mental health consumers rank their mental health as the leading health priority. In contrast, other health factors, such as physical health, accommodation, and family and friends were ranked as significantly less important health priorities [36].

While not investigated in the present study, previous studies have demonstrated that mental health consumers report significant benefits associated with having a care manager to assist with navigating the healthcare system, including establishing linkages with primary care providers [37]. However, Australian data indicate that less than half of the mental health consumers routinely visit the same GP [1]. Coupled with difficulty in communicating mental health problems with GPs [38], and a disparity between the recovery-oriented approach of the consumers and the treatment-based approach of the GP [39], this lack of continuity of care may be one factor that contributes to the continued poor physical health of mental health consumers. Future studies of CHN interventions should consider the inclusion of qualitative research examining the effect of the intervention from a consumer perspective.

Our data add little to our understanding of the incentives for and barriers to change. This is perhaps partly due to people with SMI experiencing difficulty in articulating how their physical healthcare needs are being met. Alternatively, a single consultation with the CHN may be insufficient to develop a therapeutic relationship where the consumer feels comfortable divulging this information. Another possibility is the manner by which the CHN questioned the consumer regarding incentives for and barriers to positive behavior change, interpreted the responses, and transcribed these into the case notes. The lack of detail provided in the nursing notes is described in the literature [40, 41], with nurses tending to prefer verbal communication to written [42]. In any case, providing training for the CHN in this specific area, which may be deemed as being outside usual nursing practice, may assist in circumventing this problem in future studies.

Few studies have comprehensively examined the incentives for and barriers to lifestyle interventions in the SMI population. One narrative synthesis of the relevant literature highlighted the contribution that illness symptoms and medication side effects have on lack of engagement with lifestyle interventions [43]. More recent reports [44] build on these suggestions, adding poverty and unemployment as barriers to the uptake of physical health interventions. In contrast, factors including contingency management, staff, or therapist participation in the intervention, and positive outcomes, such as weight loss, have been cited as incentives for participation in lifestyle interventions [43]. Such strategies are likely to enhance motivation toward change. While data from the present study are limited, one participant cited low motivation as a barrier to behavior change. Previous studies that have used interventions based on theories of behavior change [45, 46] have demonstrated improved motivation towards healthy behaviors, and are likely to address the incentives for and barriers to participation in a more effective manner, compared with atheoretical interventions.

In general, the reviews of paper-based participant files revealed inconsistent reporting of consultation details and poor reporting of recommendations and follow-up. In the present study, this is likely due to the use of a consultation recording form which was routinely used in the service, yet was not designed for cardiometabolic screening with accompanying health behavior change and follow-up consultations in mind. For future studies, and when integrating this service into usual care, effort must be directed towards developing clinical note-taking tools which capture the length and breadth of data required for the needs of the CHN. Further consideration should be given to the instrument’s ability to be integrated with electronic notes and remain compliant with clinical audit requirements. This will ensure easy access to, and interpretation of, the clinical notes by all staff involved in the care of mental health consumers, including clinicians, case managers, and nursing staff.

### Limitations

The findings of the present study should be interpreted with caution. Recruitment was broadly targeted at consumers with SMI. Since our original research plan was not to stratify outcomes by mental illness diagnosis, and to reduce interview burden on consumers, we did not collect diagnostic or symptom severity data. Consumers were invited to participate in the study by their consultant, case manager, or nurse. Therefore, the decision regarding the capacity to provide informed consent and participate in the study was left to the referrer. As a result, information regarding those who refused participation and the reasons for refusal are not available. One reason for the low recruitment may have been that only a small number of mental health consumers were invited to participate in the study.

Following recruitment, and prior to contact with the CHN, a number of consumers could no longer be contacted, withdrew consent, or subsequently failed to attend CHN appointments, resulting in a substantial dropout. While the study gained the full support of the mental health service, and considerable effort was directed toward increasing recruitment and retention, we acknowledge the small sample size limits the generalizability of the findings.

For a variety of reasons not directly related to the study, a proportion of consumers who attended initial appointments with the CHN were lost to follow-up. Two consumers were identified as having left the local region. Others could no longer be contacted, failed to return multiple unanswered telephone calls or respond to text messages, or did not attend follow-up appointments despite repeated attempts by the CHN to make contact. Those who did not attend follow-up were not significantly different in age, anthropometric characteristics, or number of chronic health conditions.

Our feasibility study was conducted in a regional mental health service in Queensland, Australia. The applicability of the role and our findings should be placed in the context of the healthcare service in which our trial was conducted. The reporting in clinical notes may have been hampered by the capacity of existing tools to accommodate the data, which were required for this study, and future implementation studies or clinical trials should pilot task-specific clinical note tools prior to commencement. Finally, our study is limited in that we did not identify the reasons why some consumers did not receive referrals for primary care or allied health services, despite the presence of risk factors for, or diagnosis of, chronic illness. Consistent with our views on the need for improved clinical record keeping, is identifying why consumers do not receive referrals in order to address consumer and systemic barriers to primary and allied healthcare delivery.

## Conclusions

Despite the above-mentioned limitations, the findings from the present study suggest that utilization of a CHN may be a potentially useful strategy to addressing the enduring problem of the poor physical health care of mental health consumers. The improved connectedness with primary care services and opportunity for the CHN to effect positive behavior change in mental health consumers warrants further investigation.

## Figures and Tables

**Table 1:** Summary of cardiometabolic health nurse referrals to healthcare professionals.

Healthcare service	Number of referrals made	Purpose of referral
General practitioner	8	Routine blood tests
Breast screening services	2	Mammography
Women’s Health Centre	1	Tai Chi Classes
Nutritionist	2	Nutrition counselling
Nurse-led services	9	Physical activity and nutrition counselling
